# The effect of initial support on fitness center use in new fitness center members. A randomized controlled trial

**DOI:** 10.1016/j.pmedr.2021.101605

**Published:** 2021-10-19

**Authors:** Liv Riseth, Tom Ivar Lund Nilsen, Øyvind Mittet, Aslak Steinsbekk

**Affiliations:** a Department of Public Health and Nursing, Norwegian University of Science and Technology, Post box 8905, 7491 Trondheim, Norway; b 3T- Fitness Center, Vestre Rosten 80, 7075 Tiller, Norway; c Clinic of Anaesthesia and Intensive Care, St Olavs Hospital, Trondheim University Hospital, Trondheim, Norway

**Keywords:** Fitness center, Fitness trainer bookings, Randomized controlled trial, Membership duration, Visits, Fitness center use, Telephone, E-mail

## Abstract

This provider and participant blinded parallel-group randomized controlled trial aimed to investigate if initial support given to new members via telephone and e-mail, compared to self-directed use, had an effect on booking with a fitness trainer, number of visits to the center, and membership duration. Participants included 356 new members, 174 randomized to the intervention group, and 182 to the control group. The intervention group received support to use the fitness center facilities through two phone calls and one e-mail over the first eight weeks of their membership. The control group got usual practice, which is self-directed use.

Participants in the intervention group were more likely to book at least one session with a fitness trainer during the first six months (odds ratio 1.6, 95% confidence interval (CI) 1.0–2.5). However, the intervention did not influence the number of visits (mean difference after four years −11.7 days, 95% CI −34.8 to 11.3) or time to membership termination during the follow-up period (hazard ratio 1.1, 95% CI 0.8–1.3).

In conclusion, initial support to use the fitness center facilities given to new fitness center members via telephone and e-mail increased the proportion of bookings with a fitness trainer during the first months of the membership, but it did not have an effect on the number of visits or membership termination during four years.

## Highlights

• There is a lack of research on what fitness centers do to support new members.
• The effect of initial support delivered via telephone and e-mail was investigated.
• There was an effect on bookings with a fitness trainer first six months.
• There was no effect on days with visits or membership duration during four years.
• More visits the first six months were associated with longer membership duration.

## 1 Introduction

Public reports show that in 2017, 11% of citizens within the European Union reported a fitness center membership (Commission and Eurobarometer, 2017). In Norway, the number of fitness centers has more than doubled during the past decade, from 477 in 2008 to 1228 in 2019 (Open et al., 2018, Open et al., 2019), and 30% of Norwegians aged 15 years or more had a fitness center membership in 2018 (Open et al., 2018). Thus, fitness- and sports centers where members pay to exercise constitute an essential arena for physical activity.

Although fitness center members report an intention of regular fitness center use at the time of enrollment (Garon et al., 2015), many have few visits, and some never visit the fitness center during the first membership month (Middelkamp et al., 2016, Gjestvang et al., 2020). One study found that 50 percent did not visit the fitness center the first membership month and 20 percent never attended the center during 24 months (Middelkamp et al., 2016) while another found that 37 percent were classified as regular exercisers throughout the first year of the membership (Gjestvang et al., 2020). Moreover, around 50 percent are reported to terminate their membership during the first year (Clavel San Emeterio et al., 2019, MacIntosh and Law, 2015, Emeterio et al., 2020). This mismatch between the intention at enrollment and the subsequent use of the fitness center suggests an unfulfilled potential to help member's increase their fitness center use.

We have not identified any research describing what fitness centers do to support new members to start using their services, and consequently not any studies investigating the effect of such interventions. However, as the frequency of visits, particularly during the start-up phase, has been positively associated with later use of the exercise facilities (Middelkamp et al., 2016) and longer membership duration (Clavel San Emeterio et al., 2019, Emeterio et al., 2020), getting new members to start using the fitness center in the initial phase of the membership seems worthwhile. One approach is to contact members by telephone and mail to offer initial support to start using the fitness center. Although this approach has been investigated in other settings (Goode et al., 2015, Goode et al., 2012, Fischer et al., 2019, Vandelanotte et al., 2007), it has not been investigated in a fitness center setting.

Therefore, this randomized controlled trial aimed to investigate if initial support given to new fitness center members via telephone and e-mail to use the fitness center facilities, compared to self-directed use, had an effect on booking with a fitness trainer free of extra charge, the number of visits to the center and membership duration during four years after enrollment.

## 2 Materials and methods

This was a participant and provider-blinded, parallel-group, randomized controlled trial. The inclusion was done in the first week of September 2014, and follow-up data from the membership register covered September 2014 to September 2018. The Consolidated Standards of Reporting Trials (Schulz et al., 2010) and The Template for Intervention Description and Replication (Hoffmann et al., 2014) were consulted for the reporting. There were no changes to the methods after trial commencement.

### 2.1 Setting

The study was conducted at the 3T-Fitness Center chain (www.3t.no) in Trondheim, Norway. The fitness chain had eight centers and approximately 34.000 members in 2014, and the number of inhabitants aged ≥ 16 years in Trondheim was around 165.000. The centers in this chain have a diversity of training facilities with a main workout area that consists of various exercise equipment, and all centers are staffed. In addition, seven out of eight centers offer group activities. The monthly cost for a membership in this fitness center chain was approximately 350–500 NOK (35–50 EUR) and most new members subscribe to a 12-month binding contract, which subsequently runs until terminated by the member. The front staff at each center consists of receptionists, as well as fitness trainers with minimum a bachelor's degree in physiotherapy, movement science or similar. The fitness trainers are present in the main workout area and are available for questions and guidance in relation to activities and exercises.

At the time of enrollment, all members receive information about the benefits of the membership and practical use of the fitness center by the receptionist. The usual practice at the centers is self-directed use with possibilities for help and guidance from front staff upon request. In addition, members can book a free-of-charge session with a fitness trainer and get a personalized exercise program. Members can also choose to pay for a personal trainer for closer follow-up and guidance.

### 2.2 Recruitment and inclusion criteria

The study included all persons 16 years or older that signed up for a 12-month ordinary contract with continuous monthly automatic membership renewal afterwards, who started their membership at one of the eight 3T-Fitness Centers in the City of Trondheim between September 1st and September 9th, 2014.

### 2.3 Intervention

The intervention was developed by the fitness center chain head office and delivered as planned with no changes after trial commencement. The idea was to give new members initial support via two phone calls 1–3 and 6–8 weeks after enrollment as a member and an e-mail after four weeks (Table 1), aiming to support members to use the fitness center facilities, including group activities and face-to-face support from a fitness trainer. Fitness trainers are in this fitness center chain a free service, giving the members a possibility to get a tour at the fitness center, guidance concerning exercise, a personal exercise plan, and information about group activities and use of other services.

**Table 1:** Timing, implementation, and content of the intervention to support members to start to use the facilities.

**Timing and implementation**	**Summary of the template content**
Week 1–3: Phone call from a fitness trainer	Ask if the member has started to use the fitness center and if there are any questions related to the use. - If started to use, further questions about their use in general and group activities in particular. - If not started to use, questions whether the member has used a fitness center before and if there is anything the staff could do to make it easier to get started. Inform the member about the possibility to get guidance from a fitness trainer free of extra charge and ask if the member has had guidance with a fitness trainer. - If yes, further questions about experiences with the guidance from the fitness trainer, goals set during the guidance and use of the exercise program. - If no, the value of guidance with a fitness trainer should be emphasized, highlighting the opportunity to get help, which will make it easier to reach own goals for the membership. Explore if the member has questions and what is needed to be more satisfied with the membership.Inform about the possibility for help and support from the front staff.
Week 4: E-mail sent from head office to members registered with an e-mail address	Information about guidance from a fitness trainer, how to reach physical activity goals, link to information about some examples of strength- and cardiovascular programs and popular activities at the fitness center. Furthermore, link to frequently asked questions and motivational campaigns and information about the possibility for help and support from the front staff.
Week 6–8: Second phone call from a fitness trainer	Same procedure and content as the first phone call but adjusted to the members use or not of the fitness center and guidance from a fitness trainer.

Each fitness center organized the calls for its members by using available fitness trainers, typically from one to eight at the different centers. The fitness trainers used a template to guide the content, structure, and wording during the call and received no other instructions or training. The intention was that all participants should get two calls, and therefore, two attempts to reach the members were made for each of the two phone calls, and if they did not answer, a voice message was left on the participant's phone. In the voice message, the participants were informed about the possibility of guidance with a fitness trainer and encouraged to contact the fitness center if they had questions about use of the fitness center.

The control group received usual practice, which is self-directed use with possibilities for help and guidance from staff upon request.

### 2.4 Ethical considerations

The study was conducted in accordance with the World Medical Association Declaration of Helsinki – Ethical Principles for Medical Research Involving Human subjects. Data were anonymized and only available to the authors after anonymization. According to Norwegian law, only health research, which this study is not, is assessed by The Regional Committee for Medical and Health Research Ethics. A request was sent to the Norwegian Centre for Research Data, which assesses privacy and data protection. They replied that registration was not required as no identifying or sensitive information was used. The study was considered to have low or no risk for the participants due to the nature of the intervention (phone calls and e-mail), and there was no collection of sensitive data.

### 2.5 Outcome measures

To obtain outcome data, the head office of the fitness center chain sent a list with the membership ID numbers for those meeting the inclusion criteria to the provider of the membership register, who returned a file with membership and activity data. To ensure the quality of the data, an employee in the head office of the fitness center chain did random checks of the data file. Finally, de-identified data on age, sex, and timestamps for each visit, date of membership termination and date for appointment for individual guidance with a fitness trainer was delivered to the first author.

The outcomes were number of days the participants had been registered with one or more visits, having made a booking with a fitness trainer free of extra charge at three months, six months and four years after enrollment, and membership duration measured as proportion who terminated their membership during four years as well as time to membership termination. There were no changes to trial outcomes after the trial commenced.

### 2.6 Sample size

The Sampsi procedure in STATA for comparison of two means was used for sample size calculations. This suggested that 143 persons in each group was sufficient to detect a mean difference at three months of two visits between the groups using an assumed standard deviation of 6 visits (i.e. standard deviation three times larger than the mean difference), alpha level of 0.05 and a power of 80%. Based on the enrollment history from previous years, it was expected that approximately 350 new members meeting the inclusion criteria would enroll during the first week of September 2014.

### 2.7 Randomization and allocation

A list with random numbers generated in Excel was delivered to an employee in the head office not affiliated with the study, who consecutively added the name and contact information of new members as they enrolled in the one-week recruitment period. Then the list was sorted on the random number and the 175 participants with the smallest numbers (first in the list) were assigned to the intervention group, and the remainder were assigned to the control group. Each center then received a list of the new members in the intervention group enrolled at their center, which was used to conduct the phone calls. Another employee in the head office used the list to send out the e-mail.

### 2.8 Blinding

Neither the participants nor the staff who performed the intervention were informed that the phone calls and e-mail were part of a research study. All new members of the fitness center signed a membership contract upon enrollment where they acknowledge that the fitness center could contact them with information about the membership and use of the fitness center. Furthermore, the staff at the fitness centers were not told that the phone calls and e-mail constituted the intervention in a study, but that this was a test of new routines and a possible solution to help members to get started in the initial phase of their membership. This is common practice for new initiatives within this fitness center chain. The outcome data were automatically registered in the membership registry and could not be influenced by anyone in the research group.

### 2.9 Statistical methods

All analyses were done according to the intention to treat principle and included all participants initially randomized to the intervention or control group. Mean differences in number of days with visits between the groups were estimated using linear regression. Odds ratios (OR) for having booked with a fitness trainer was estimated using logistic regression. Differences in membership duration was displayed as a Kaplan-Meier curve, and the relative difference between the groups was assessed as a hazard ratio (HR) using Cox regression. Due to former research reporting sex and age differences in membership duration (Emeterio et al., 2020), all models included adjustments for sex (woman, man) and five age groups categorized to have approximately same number in each group, which gave groups from 16 to 19, 20–24, 25–29, 30–39, and 40–76 years. The precision of estimated effects was assessed by a 95% confidence interval (95% CI).

Additional analyses were conducted to explore whether sex, age, frequency of visits, and having booked with a fitness trainer during the first six months were associated with membership duration in the whole sample. The first six months' average number of visits were categorized into four groups with an approximately equal number of participants (quartiles), ranging from 0 to 12 visits in the first category, 13–24 visits in the second, 25–48 visits in category three, and the last included 49–105 visits. Differences in membership duration were estimated as HRs with 95% CI using Cox regression. In addition, associations with age were adjusted for sex (woman, man), and fitness trainer and number of visits were adjusted for the five age-groups shown above and sex (woman, man).

## 3 Results

**Fig. 1:**
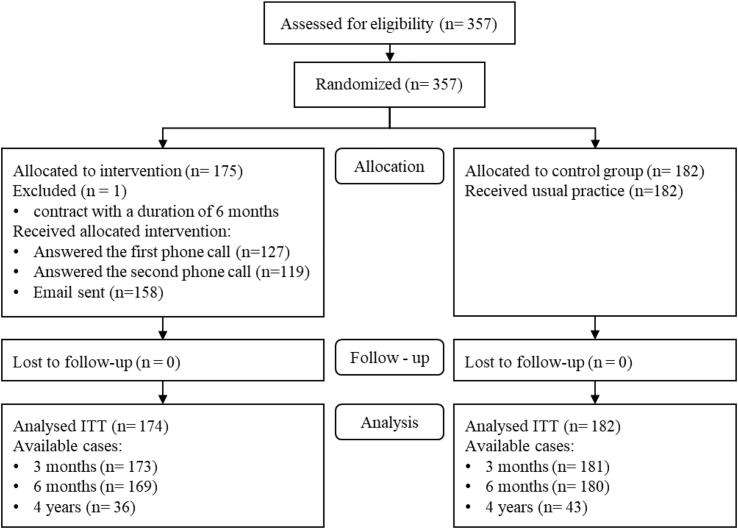
Participant flow diagram through the study.

A total of 357 new members were initially included in the trial (Fig. 1). One participant with a six-month instead of a 12-month contract was wrongly included, but was excluded after randomization and not included in the analysis. This left 356 participants for the analyses (174intheinterventiongroupand182inthecontrolgroup). The groups were similar at baseline with respect to sex and age (Table 2).

**Table 2:** Participant characteristics at baseline for the intervention and control group (N = 356).

Characteristic	Intervention group (n = 174)	Control group (n = 182)
Sex, n (%)		
Female	111 (63%)	113 (62%)
Male	63 (37%)	69 (38%)
Mean age, years (SD)	30 ()	29 ()
Age groups, n (%)		
16 to 19	27 (15%)	32 (18%)
20 to 24	48(28%)	62 (34%)
25 to 29	35 (20%)	31(17%)
30 to 39	30 (17%)	27 (15%)
40 to 76	34 (20%)	30 (17%)

### 3.1 Implementation of the intervention

The first phone call reached 127 out of the 174 (73%) participants in the intervention group and the second phone call reached 119 (68%). The e-mail was sent to the 158 (91%) participants who had registered their e-mail address at the fitness center.

### 3.2 Fitness trainer bookings

The intervention had an effect on the proportion of participants who booked a fitness trainer during the first three (OR 1.8, 95% CI 1.1–2.7) and six (OR 1.6, 95% CI 1.0–2.5) months (Table 3).

**Table 3:** Effect of the intervention on booking with a free fitness trainer during three and six months after baseline.

	Booked with a fitness trainer			
Period	Yes, n (%)	No, n (%)	Crude OR	Adjusted OR(95% CI)^a^
3 months				
Control	59 (32%)	123 (68%)	1.0	1.0 (Reference)
Intervention	78 (45%)	96 (55%)	1.7	1.8 (1.1–2.7)
6 months				
Control	67 (37%)	115 (63%)	1.0	1.0 (Reference)
Intervention	83 (48%)	91 (52%)	1.6	1.6 (1.0–2.5)

Abbreviations: OR = odds ratio. CI = confidence interval

a Adjusted for age (16–19, 20–24, 25–29, 30–39 and ≥ 40 years) and sex (man, woman)

### 3.3 Number of visits

The intervention did not increase the number of visits to the fitness center (Table 4). The mean number of days with visits from baseline to six months was 29 days in the intervention group and 32 days in the control group (mean difference −2.3 days, 95% CI −7 to 2.5), while it was 98 compared to 109 days after four years (mean difference after 4 years −11.7 days, 95% CI −34.8 to 11.3).

**Table 4:** Mean number of days with visits and mean difference between intervention and control group.

Time from baseline	Mean number of days with visits (SD)			Mean difference between groups	
	Intervention	Control		Crude difference	Adjusted difference (95% CI)^a^
3 months	16.8 (12.4)	18.3 (13.4)		− 1.4	−1.0 (-3.7 to 1.6)
6 months	29.2 (23.1)	32.0 (23.2)		− 2.8	−2.3 (-7 to 2.5)
4 years	98.1 (107.1)	108.9 (114.2)		− 10.8	− 11.7 (-34.8 to 11.3)

Abbreviations: SD = standard deviation; CI = confidence interval

a Adjusted for age (16–19, 20–24, 25–29, 30–39 and ≥ 40 years) and sex (man, woman)

### 3.4 Membership duration

**Fig. 2:**
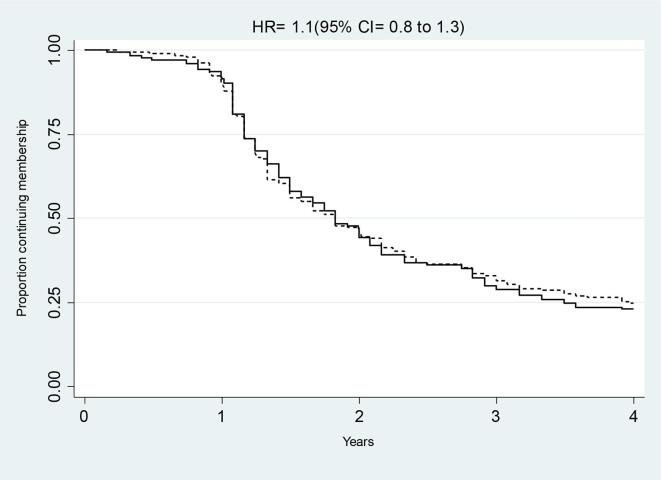
Kaplan-Meier survival curve and hazard ratio for membership termination during four years.

The proportion of persons terminating their membership during the four-year follow-up period was also similar between the intervention (79%) and control (76%) group. Furthermore, there was no difference in time to membership termination between the groups during the four years (HR 1.1, 95% CI 0.8–1.3) (Fig. 2).

### 3.5 Additional analyses

There was no evidence that booking with a fitness trainer or sex was associated with membership duration during four years in the whole sample (Table 5). However, age was inversely associated with time to membership termination, where members aged ≥ 40 years had a HR of 0.3 (95% CI 0.2–0.4) compared to those aged 20–24 years. Also, number of visits the first six months showed an inverse association with membership termination, where participants with ≥ 49 visits during four years (at least two visits each week) had a HR of 0.6 (95% CI 0.4–0.9) compared to members with 12 visits or less.

**Table 5:** Additional analyses of factors associated with membership terminations during four years.

Variables	Number at baseline	Number and proportion terminating membership during four years	Crude hazard ratio	Adjusted hazard ratio (95% CI)^a^
Sex				
Female	224	168 (75%)	1.0 (Reference)	
Men	132	103 (78%)	1.0 (0.8–1.4)	
Age				
16–19 years	59	47 (80%)	0.7	0.7 (0.5–0.9)
20–24 years	110	86 (78%)	1.0	1.0 (Reference)
25–29 years	67	41 (61%)	0.6	0.6 (0.4–0.9)
30–39 years	57	37 (65%)	0.5	0.5 (0.4–0.8)
40–76 years	64	27 (42%)	0.3	0.3 (0.2–0.4)
Booking with fitness trainer				
No	206	138 (67%)	1.0	1.0 (Reference)
Yes	150	100 (67%)	1.1	1.1 (0.9–1.4)
Number of visits				
0–12 visits	91	78 (86%)	1.0	1.0 (Reference)
13–24 visits	84	58 (69%)	0.7	0.7 (0.5–1.0)
25–48 visits	104	75 (72%)	0.7	0.7 (0.5–1.0)
49–105 visits	78	61 (78%)	0.7	0.6 (0.4–0.9)

Abbreviations: CI = confidence interval

a Associations with age are adjusted for sex (man, woman); fitness trainer bookings and visits are adjusted for age (16–19, 20–24, 25–29, 30–39 and ≥ 40 years) and sex (man, woman)

### 3.6 Harms

No harms or unintended effects was observed or reported during the study

## 4 Discussion

New fitness center members who received initial support to use the fitness center, delivered through telephone and e-mail contact, were more likely to book appointments with a fitness trainer during six months of follow-up compared to the control group who received usual practice. The intervention had no effect on number of days with visits to the fitness center or on membership duration during the four-year follow-up period.

Since the intervention increased the proportion who booked with a fitness trainer, it is likely that the content of the initial support influenced the start-up behavior and made members aware that this service was available. However, even though the intervention increased the likelihood to book with a fitness trainer, there was no effect on the number of visits in the same time period or membership duration during four years. The latter was also supported by additional analyzes, where booking with a fitness trainer was not associated with membership duration.

The lack of effect of the intervention should be viewed in light of the limited possibility to individualize the communication with the members within the standardized template for content, structure and wording in the phone calls. However, individualized interventions applying different behavior change techniques, such as goal setting, have been found to increase physical activity (Fischer et al., 2019, Nigg et al., 2008, Howlett et al., 2019). Therefore, the effect of a more individualized approach that includes behavior change techniques could be worth examining also for new members of fitness centers.

The delivery method included one e-mail and two phone calls and no face-to-face support. Previous research has shown that support through e-mail (Plotnikoff et al., 2010), telephone (Goode et al., 2012), or face-to-face (Richards et al., 2013) can change behavior, but differences between and combinations of these methods have been less explored. An intervention study indicates telephone support to be as effective in increasing physical activity as face-to-face support (Opdenacker and Boen, 2008). However, a review concluded that there was insufficient evidence to assess whether it is effect differences between face-to-face interventions or remote and web 2.0 approaches at promoting physical activity (Richards et al., 2013). Second, the quantity of the intervention was limited to two phone calls and one e-mail. A previous study found that a telephone delivered intervention with three calls increased the participant's attention towards the printed material in the study, but had no effect on the targeted physical activity (walking) (Humpel et al., 2004). However, a randomized trial with up to 12 calls found higher physical activity levels than a minimal intervention with written physical activity recommendations (Fischer et al., 2019). Frequent and focused follow-up through remote or web interventions has effectively encouraged the uptake of physical activity (Foster et al., 2013;CD010395.), as have other interventions with varying modes and frequency of contact (Goode et al., 2012, Vandelanotte et al., 2007). Thus, the optimal delivery mode and number of contact points need further investigation.

Another aspect is how the intervention was experienced. Since the intervention was blinded to the participants, the fitness center members who received the intervention (i.e., phone calls and e-mail) were not informed about this contact in advance. This type of contact was probably not expected when signing up for a membership, and one could speculate that some could receive this as an intrusion or sale, not support. In a randomized trial showing effect, the participants were aware that they could be contacted by telephone when they signed up for the study (Fischer et al., 2019). It was not collected data on how the phone calls were perceived, but some of the fitness trainers performing the phone calls said they experienced that a few members reacted negatively to the calls. Privacy, personal freedom and not wanting to be bothered at home are some attitudes toward telephone surveys reported (Kolar and Kolar, 2008). To avoid such reactions, the fitness centers could ask new members if they want to be contacted or not.

After 12 months when the compulsory part of the membership contract ended, there was a steady decline in membership retention and more than half had terminated their membership after two years, similar to other studies (Clavel San Emeterio et al., 2019, Emeterio et al., 2020). The additional analysis showed that time to membership termination was shortest for members with fewest visits during the first six months (less than one day with visit every other week). In a study from Spain, more than eight visits a month was associated with the lowest odds for termination (Clavel San Emeterio et al., 2019). This support the assumption that increased visits in the start-up phase of a membership would lead to longer membership duration. Even though many members terminated their membership, more than 20% continued as a member over four years in the present study. This group of long-term members could give valuable insight into factors that influence membership use and duration in future studies.

### 4.1 Strengths and limitations

This is the first randomized controlled trial investigating the effects of initial support given to new members via telephone and e-mail on use of the fitness center facilities. Considering the Revised Cochrane risk-of-bias tool for randomized trials (Higgins et al., 2019), the study results are evaluated to be at low risk of bias. A main strength of this study was the randomized controlled design, blinding of participants and those delivering the intervention, and inclusion of the number of participants indicated by the sample size calculation. However, the variation in number of visits was larger than expected, resulting in unprecise estimates of differences between the groups.

The outcome variables and the period analyzed were pre-planned. There were no baseline differences between the groups, indicating a successful randomization process. Use of register data ensured that outcome measures were available for all participants and provided a complete follow-up. The study was conducted in a real-life setting and thus likely to be generalizable to similar fitness centers. Unfortunately, this study did not measure the member's physical activity behavior, and their physical activity levels are therefore unclear.

## 5 Conclusion

This study reports data from a randomized controlled trial showing that new fitness center members who receive two initial phone calls and one e-mail to support them to use the fitness center were more likely to book one or more fitness trainer appointments during the first six membership months. However, initial telephone and e-mail support had no effect on number of days with visits to the fitness center or on membership duration during the four-year follow-up period.

There is a clear need for future high-quality studies building upon this study. Future research could focus on investigating whether other delivery modes and number of contact points are more effective.

## 6 Availability of data and material

The anonymized datafiles and trial protocol are available from the corresponding author on reasonable request. The trial protocol was not published before trial commencement.

## Funding

Liv Riseth is an Industrial Ph.D. candidate funded 50/50 by 3 T-Fitness Center and The Research Council of Norway (grant number 239657) and was employed at 3 T-Fitness Center from January 2013 until June 2018.

Registration: ClinicalTrials.gov trial (NCT03989804)

### CRediT authorship contribution statement

**Liv Riseth:** Conceptualization, Methodology, Formal analysis, Visualization, Investigation, Funding acquisition, Project administration, Writing – original draft, Writing – review & editing. **Tom Ivar Lund Nilsen:** Conceptualization, Methodology, Formal analysis, Supervision, Visualization, Writing – original draft, Writing – review & editing. **Øyvind Mittet:** Conceptualization, Methodology, Investigation, Project administration, Writing – review & editing. **Aslak Steinsbekk:** Conceptualization, Methodology, Formal analysis, Project administration, Funding acquisition, Supervision, Visualization, Writing – original draft, Writing – review & editing.

## Declaration of Competing Interest

The authors declare that they have no known competing financial interests or personal relationships that could have appeared to influence the work reported in this paper.
